# Investigating the Role of Mitochondrial Haplogroups in Genetic Predisposition to Meningococcal Disease

**DOI:** 10.1371/journal.pone.0008347

**Published:** 2009-12-17

**Authors:** Antonio Salas, Laura Fachal, Sonia Marcos-Alonso, Ana Vega, Federico Martinón-Torres, Grupo de investigación ESIGEM (Estudio Sobre la Influencia Genética en la Enfermedad Meningocócica)

**Affiliations:** 1 Unidade de Xenética, Departamento de Anatomía Patolóxica e Ciencias Forenses and Instituto de Medicina Legal, Universidade de Santiago de Compostela, Santiago de Compostela, Galicia, Spain; 2 Fundación Pública Galega de Medicina Xenómica, Hospital Clínico Universitario de Santiago, Santiago de Compostela, Galicia, Spain; 3 Servicio de Pediatría, Hospital Universitario de A Coruña, A Coruña, Galicia, Spain; 4 Servicio de Críticos, Intermedios y Urgencias Pediátricas, Hospital Clínico Universitario de Santiago, Santiago de Compostela, Galicia, Spain; 5 Grupo Gallego de Genética, Vacunas e Investigación Pediátrica, Instituto de Investigación Sanitaria de Santiago, Santiago de Compostela, Galicia, Spain; Institut Pasteur, France

## Abstract

**Background and Aims:**

Meningococcal disease remains one of the most important infectious causes of death in industrialized countries. The highly diverse clinical presentation and prognosis of *Neisseria meningitidis* infections are the result of complex host genetics and environmental interactions. We investigated whether mitochondrial genetic background contributes to meningococcal disease (MD) susceptibility.

**Methodology/Principal Findings:**

Prospective controlled study was performed through a national research network on MD that includes 41 Spanish hospitals. Cases were 307 paediatric patients with confirmed MD, representing the largest series of MD patients analysed to date. Two independent sets of ethnicity-matched control samples (CG1 [*N* = 917]), and CG2 [*N* = 616]) were used for comparison. Cases and controls underwent mtDNA haplotyping of a selected set of 25 mtDNA SNPs (mtSNPs), some of them defining major European branches of the mtDNA phylogeny. In addition, 34 ancestry informative markers (AIMs) were genotyped in cases and CG2 in order to monitor potential hidden population stratification. Samples of known African, Native American and European ancestry (*N* = 711) were used as classification sets for the determination of ancestral membership of our MD patients. A total of 39 individuals were eliminated from the main statistical analyses (including fourteen gypsies) on the basis of either non-Spanish self-reported ancestry or the results of AIMs indicating a European membership lower than 95%. Association analysis of the remaining 268 cases against CG1 suggested an overrepresentation of the synonym mtSNP G11719A variant (Pearson's chi-square test; adjusted *P*-value = 0.0188; OR [95% CI] = 1.63 [1.22–2.18]). When cases were compared with CG2, the positive association could not be replicated. No positive association has been observed between haplogroup (hg) status of cases and CG1/CG2 and hg status of cases and several clinical variants.

**Conclusions:**

We did not find evidence of association between mtSNPs and mtDNA hgs with MD after carefully monitoring the confounding effect of population sub-structure. MtDNA variability is particularly stratified in human populations owing to its low effective population size in comparison with autosomal markers and therefore, special care should be taken in the interpretation of seeming signals of positive associations in mtDNA case-control association studies.

## Introduction

The clinical presentation of meningococcal infections caused by *Neisseria meningitidis* is highly diverse. Invasive infections usually result in meningococcaemia, meningitis, or both [1], [2]. Meningococcal disease (MD) remains one of the most important infectious causes of death in industrialized countries [1], [2]. Mortality rates of patients with severe meningococcal sepsis may reach up to 40–50%, and those that survive may have severe complications that often require amputation and/or skin grafting [2]. The bacterium, *Neisseria meningitidis*, is only found in human hosts, and asymptomatic carriage can exceed 70% during outbreak situations [3]. The pathophysiology consists of a complex interaction of bacterial and host factors, triggered mainly by the release of endotoxin [3]. Individual response to meningococcal lipopolysacharides involves also non-immunological mechanisms and the activation of three main cascade reactions, the complement system, the coagulation and fibrinolysis pathways, and the inflammatory reaction mediated by different cytokines and chemokines [4]. Besides, many studies already support the idea that genetics plays an important role in MD (see [5] for a recent review). In this sense, we might hypothesize that mitochondrial genetic background could be one of these host genetic factors determining the response to *Neisseria meningitides* infection and the individual course of meningococcal disease.

Mitochondrions contain multiple copies of mtDNA genomes. Each double-stranded circular mtDNA molecule consists of approximately 16569 base pairs (bps) encoding 37 genes: thirteen polypeptides (subunits of Complex I, III, and IV plus the ATP synthase complex), two ribosomal RNAs (rRNAs), and 22 transfer RNAs (tRNAs). The mtDNA is inherited through the matriline as a haplotypic block. In the jargon of population geneticists, clusters of closely-related mtDNA haplotypes are known as haplogroups (hgs). During the last two decades, population geneticists have comprehensively investigated the evolutionary patterns of worldwide mtDNA hgs, so that nowadays there is good knowledge of the global mtDNA phylogeny and the polymorphisms defining main and minor branches of the tree (basal motifs) (see e.g. [6], [7], [8], [9], [10], [11], [12], [13], [14], [15]), as well as positional-mutation rates [16]. On the other hand, several sporadic or inherited mtDNA mutations are responsible for a number of mtDNA diseases (Leber's hereditary optic neuropathy [LHON], Leigh syndrome, etc) [17], [18]. In addition, genetic variation of mtDNA has also been linked to several multi-factorial diseases (e.g. [19], [20]) although some of their conclusions have been questioned (e.g. [7], [11], [21]).

The mtDNA variation has already been investigated in several infectious diseases. For instance, in an adult AIDS clinical trials group study, Hulgan et al. [22] claimed that the typically European hg T was involved in peripheral neuropathy during antiretroviral therapy. The results of Hendrickson et al. [23] suggest that mitochondrial genes are important indicators of AIDS disease progression in HIV-1 infected persons. Houshmand et al. [24] did not detect statistical significant differences between tuberculosis patients (*N* = 54) and a control group (*N* = 256) in a case-control study. MtDNA predisposition to sepsis has also been investigated in several studies. Thus, Baudouin et al. [25], by analysing 150 individuals who were sequentially admitted to an intensive care unit in the UK and 542 age-matched controls, determined that mtDNA hg H was a strong independent predictor of outcome during severe sepsis, conferring a 2.12-fold (95% CI 1.02–4.43) increased chance of survival at 180 days compared with individuals without hg H. More recently, Yang et al. [26] claimed that hg R can predict survival advantage in severe sepsis, a conclusion derived from the prospective analysis of 181 Han patients recruited in an intensive care unit.

There are major causes of type I errors in population-based association studies, such as hidden population sub-structure or inadequate correction for multiple testing [27], [28], [29], [30], [31], [32], [33]. The confounding effect of population sub-structure is particularly problematic in mtDNA studies because variation is strongly stratified in populations owing to its smaller effective population size in comparison with autosomal markers. For instance, it is well known from population genetic studies that several mtDNA variants are strongly stratified in the Iberian Peninsula, such as hg V, H1a, H3 and H5 that are highly prevalent in the Franco-Cantabrian refugee [6], [34], [35], [36]; therefore, the Spanish population cannot be considered a single homogeneous population. Multi-centric studies involving dozens of laboratories and different medical specialists can help to add extra ‘noise’ to the sampling collection; for instance, the use of subjective criteria to assign ethnic classes to patients or assignment of ethnicity based on self-reported ancestry [37], [38] might contribute to increase stratification in mtDNA studies.

The present study aims to evaluate the potential pathogenic role of well-known mtDNA variants in MD. In contrast with most typical mtDNA case-control association studies, the additional goal of the present project was to further investigate the effect of confounding factors (such as stratification) in determining the apparent association of particular variants with the disease.

## Results

### Ancestry Assessment of Patients

**Figure 1:**
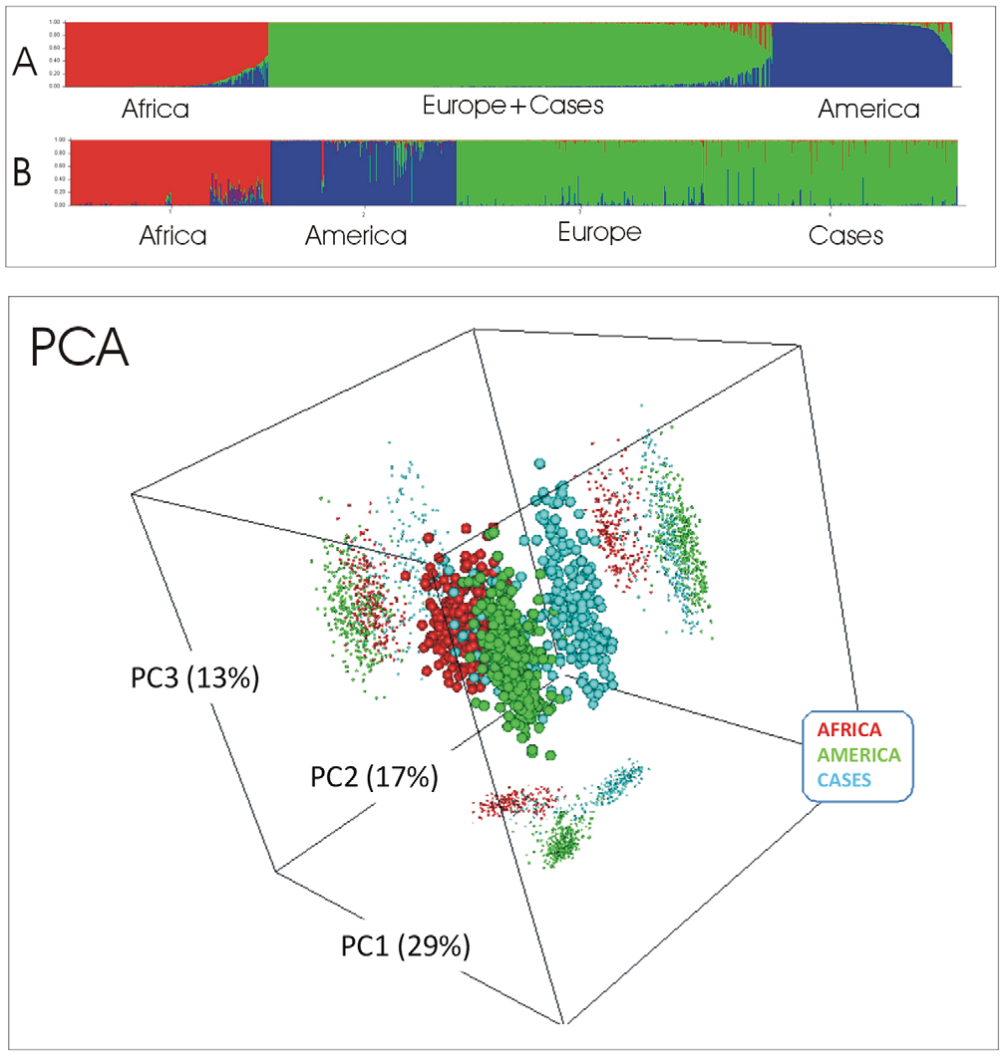
Analysis of population sub-structure in MD cases. On the left, analysis of population structure in CEPH panel samples (including 225 Africans, 208 Native Americans and 278 Europeans; see text for more details), and 307 meningococcal patients, based on a 34-plex autosomal AIMs for the assignment of ancestral origin with grouping values of *K* = 3. Top panel (A) is the representation of membership of the samples as originally introduced in the analysis; the bottom panel (B) shows the same samples sorted by membership values. On the right, Principal Component Analysis of the same samples used in Text S1. PC1, PC2, and PC3 stand for principal components one, two and three, respectively; in rounded brackets is the amount of variation accounted by each component.

Analysis of ancestry in patients was assessed by use of a classification group of population samples of known African, American, and European ancestry. As indicated in Figure 1, most of the cases have European ancestry; the average European membership in cases was 95% (SD: 10%), virtually equal to the values obtained for a typical European dataset. PCA plot (Figure 1) indicates that meningococcal patients clearly cluster in a single group, as evidenced also by the African and American samples. The amount of variation accounted by the PC1, PC2, and PC3 was ∼29%, ∼17%, and 13% respectively; ∼59% in total.

### Evaluating the Potential Association of mtDNA Haplogroups and Clinic-Pathological Variants

A total of 39 individuals were eliminated from the main statistical analyses (including fourteen gypsies) on the basis of non-Spanish self-reported ancestry or based on the results of AIMs indicating European membership lower than 95%. Association analysis was performed for the remaining 268 cases against CG1 (*N* = 917). The power to detect odds ratios as low as 1.6 for mtSNPs and considering an average population minimum allele frequency (MAF) of 20% (lower than the average MAF in CG1) was 92%. Pearson's chi-square or Fisher's exact tests were used to assess association. We adjusted for multiple tests by implementing a permutation procedure as done in [21] and using 20,000 permutations. Association analysis of cases with CG1 suggested an overrepresentation of the synonym mtSNP G11719A variant (Pearson's chi-square test; adjusted *P*-value = 0.0188; OR [95% CI] = 1.63 [1.22–2.18]) with respect to CG1 (Table 1). The logical way to confirm this positive association is to explore a second independent sample of patients. Because, however, the prevalence of the MD is low (in contrast with other common complex diseases), recruitment of a second independent sample was not possible. The present study is in fact, to our knowledge, the largest series of MD patients analysed to date in genetic studies. The alternative way to replicate the association of G11719A variant with the disease is to explore a second sample of controls. Therefore, cases were compared against a second series of ethnicity-matched controls (CG2; *N* = 616); the power to detect ORs as low as 1.6 for mtSNPs (MAF = 20%) using CG2 was 88% (Table 1). The association of G11719A observed in comparison of cases and CG1 could not be replicated with CG2. Instead, the A4769G variant appears as statistically significant, although marginal (Pearson's chi-square test; adjusted *P*-value = 0.0469) given a nominal value of significance of α = 0.05.

**Table 1:** Pearson's chi-square test for cases *versus* CG1 and CG2.

				MD cases versus CG1					MD cases versus CG2				
SNP	rCRS ref.	MAF cases	MA	MAF	Chi2 exact	*P*-value	adjusted *P*-value	OR (95% CI)	MAF	Chi2 exact	*P*-value	adjusted *P*-value	OR (95% CI)
G3010A	G	0.28	A	0.29	1.2372	0.266	0.9918	0.83 (0.61–1.15)	0.28	0.3997	0.527	1	0.90 (0.64–1.25)
T3197C	T	0.06	C	0.06	0.0356	0.85	1	0.95 (0.53–1.68)	0.10	4.0081	0.045	0.532	1.78 (1.01–3.14)
G3915A	G	0.02	A	0.02	0.8348	0.361	0.999	0.57 (0.17–1.94)	0.04	4.8407	0.028	0.374	0.28 (0.08–0.94)
C3992T	C	0.01	T	0.01	0.1432	0.705	1	0.78 (0.22–2.77)	0.01	0.0008	0.977	1	0.98 (0.25–3.82)
T4216C	T	0.17	C	0.18	0.0647	0.799	1	0.95 (0.66–1.37)	0.16	0.1798	0.672	1	0.92 (0.62–1.35)
T4336C	T	0.02	C	0.03	2.3434	0.127	0.8886	0.40 (0.12–1.35)	0.04	4.8627	0.027	0.3628	0.28 (0.08–0.94)
A4529T	A	0.02	T	0.02	0.1596	0.69	1	1.21 (0.47–3.10)	0.00	10.2116	0.004	0.0659	14.04 (1.68–117.18)
G4580A	G	0.04	A	0.04	2.8558	0.091	0.7941	0.60 (0.33–1.09)	0.03	4.5172	0.034	0.4381	0.49 (0.25–0.96)
A4769G	A	0.02	A	0.02	0.0002	0.989	1	0.99 (0.36–2.72)	0.00	11.2448	0.003	0.0469	–
A4793G	A	0.00	G	0.00	7.1652	0.007	0.1121	–	0.01	0.0735	0.786	1	0.80 (0.16–4.00)
T6776C	T	0.08	C	0.09	0.9034	0.342	0.9988	0.77 (0.46–1–32)	0.04	2.4415	0.118	0.8732	1.62 (0.90–3.01)
C7028T	C	0.47	T	0.49	6.1755	0.013	0.2	1.42 (1.08–1.87)	0.45	1.3431	0.246	0.9906	1.19 (0.89–1.59)
G8994A	G	0.02	A	0.02	2.3745	0.123	0.8827	1.90 (0.83–4.35)	0.02	2.6354	0.105	0.8395	2.10 (0.84–5.22)
G9055A	G	0.06	A	0.06	2.7059	0.1	0.8244	1.53 (0.92–2.55)	0.10	0.1954	0.658	1	0.89 (0.54–1.48)
A10398G	A	0.19	G	0.18	1.4671	0.226	0.9835	1.23 (0.88–1.73)	0.20	0.4369	0.509	1	1.13 (0.79–1.61)
C10400T	C	0.01	T	0.01	2.6015	0.107	0.8459	2.30 (0.81–6.53)	0.01	2.2218	0.136	0.9139	2.32 (0.74–7.27)
T10463C	T	0.10	C	0.10	0.0700	0.791	1	1.06 (0.68–1.67)	0.09	0.6038	0.437	0.9999	1.21 (0.74–1.97)
C10873T	C	0.03	C	0.02	4.9968	0.025	0.3374	0.46 (0.22–0.92)	0.04	0.6441	0.422	0.9999	0.75 (0.38–1.51)
G11719A	G	0.41	A	0.38	10.7820	0.001	0.0188	1.63 (1.22–2.18)	0.47	0.3430	0.558	1	1.09 (0.82–1.46)
A12308G	A	0.17	G	0.18	0.6261	0.429	0.9998	0.86 (0.59–1.25)	0.24	7.7546	0.006	0.0927	0.59 (0.40–0.86)
C12705T	C	0.09	T	0.08	6.5570	0.01	0.1549	1.75 (1.14–2.71)	0.07	7.1341	0.010	0.1533	1.89 (1.18–3.03)
G13708A	G	0.09	A	0.09	0.6741	0.412	0.9994	0.81 (0.48–1.35)	0.09	0.5517	0.458	1	0.82 (0.48–1.39)
A13966G	A	0.02	G	0.01	3.6348	0.057	0.6202	2.35 (0.95–5.81)	0.02	0.4319	0.511	1	1.34 (0.56–3.24)
C14766T	C	0.44	C	0.43	4.2540	0.039	0.4805	1.33 (1.01–1.75)	0.48	0.1177	0.752	1	1.05 (0.79–1.40)
T16519C	T	0.33	T	0.33	0.4574	0.499	1	1.11 (0.82–1.49)	0.35	1.3385	0.247	0.9911	1.20 (0.88–1.63)

rCRS: allele in the revised Cambridge Reference Sequence (rCRS) [55]; MA: allele with the lowest frequency; MAF: minimum allele frequency computed on control individuals; Adjusted *P*-value: adjustment of chi-square *P*-values was carried out with a permutation-based approach; number of permutations = 20,000; OR (95%CI): ORs were computed with the rCRS allele as a reference.

Analysis of population mtDNA data obtained from healthy individuals representing several Spanish geographical regions supports the spurious nature of the seeming positive association found in cases *versus* CG1 for the polymorphism G11719A (Text S1). The seeming association could be most likely due to the confounding effect of population stratification; in particular, the frequency of G11719A in CG1 is unusually low (Text S1). Other consideration about the harmful effect of population stratification in mtDNA case-control disease studies are commented in Text S1.

Analysis of association between hg status of patients and controls was also carried out (Table 2).The best *P*-value was obtained for hg R (0.005), which is however non statistically significant when correcting for multiple test using Bonferroni (adjusted significant value α =  0.00058).

**Table 2:** Pearson's chi-square test for the hg status of cases *versus* CG1 and CG2.

		HG status in cases versus CG1				HG status in cases versus CG2			
HG	Freq. Cases	Chi2 exact	*P*-value	OR (95% CI)	Freq. CG1	Chi2 exact	*P*-value	OR (95% CI)	Freq. CG2
H	0.41	5.2881	0.021	1.38 (1.04–1.82)	0.41	0.9834	0.321	1.16 (0.87–1.55)	0.45
H1	0.20	0.0485	0.826	0.96 (0.68–1.35)	0.20	0.0453	0.831	1.04 (0.73–1.48)	0.21
H3	0.07	1.1113	0.292	1.33 (0.78–2.26)	0.09	1.2397	0.266	0.71 (0.39–1.30)	0.05
HV	0.47	2.4663	0.116	1.24 (0.95–1.63)	0.53	0.0086	0.926	1.01 (0.76–1.35)	0.48
I	0.02	0.0454	0.831	1.11 (0.41–3.01)	0.02	5.6451	0.018	0.17 (0.04–0.89)	0.00
J	0.06	1.1921	0.275	1.36 (0.78–2.39)	0.08	0.6421	0.423	1.27 (0.71–2.29)	0.07
J1	0.04	1.0250	0.311	1.39 (0.73–2.63)	0.06	1.0010	0.317	1.40 (0.72–2.73)	0.06
K	0.08	1.4794	0.224	0.73 (0.44–1.22)	0.06	0.0738	0.786	1.07 (0.64–1.80)	0.09
K1	0.07	1.5110	0.219	0.71 (0.41–1.23)	0.05	0.1968	0.657	1.13 (0.65–1.96)	0.08
R	0.87	8.0561	0.005	1.87 (1.21–2.90)	0.93	7.1341	0.008	1.89 (1.18–3.03)	0.93
R0	0.50	4.3115	0.038	1.33 (1.02–1.75)	0.58	0.0885	0.766	1.04 (0.78–1.39)	0.51
T	0.10	0.1017	0.750	0.93 (0.58–1.47)	0.09	0.4751	0.491	0.84 (0.51–1.38)	0.08
TJ	0.16	0.2749	0.600	1.10 (0.76–1.60)	0.17	0.0008	0.978	1.01 (0.68–1.49)	0.16
U	0.17	0.0244	0.876	1.03 (0.72–1.48)	0.18	4.9115	0.027	1.51 (1.05–2.18)	0.24
U5	0.04	0.6841	0.408	1.31 (0.69–2.49)	0.06	7.6008	0.006	2.39 (1.26–4.51)	0.10
V	0.06	2.8237	0.093	0.60 (0.33–1.09)	0.04	4.4651	0.035	0.50 (0.26–0.96)	0.03
W	0.01	0.2851	0.593	0.93 (0.23–2.34)	0.01	0.1928	0.661	0.76 (0.22–2.61)	0.01

The test was carried out for those (sub)hgs with frequences above 5% in the control groups and also the well-known branches of the West European/Iberian phylogeny I, V, and W. Note that hg frequencies were inferred using the information from the whole haplotype available; this is why for instance, the frequency of hg R0 does not match with the frequency of G11719A.

Among the several clinical variables analysed in the present study (Table 3), acute respiratory distress syndrome (ARDS) was found to be strongly associated with hg U (multinomial logistic regression; un-adjusted *P*-value = 0.0025) being a strong predictor of increasing risk to ARDS (OR [95% CI] = 12.58 [2.41–64.93]); however, this association was lost when we adjusted the model using Bonferroni's correction and considered the full set of hypotheses tested in the study.

**Table 3:** Summary of demographic and clinical data of the children included in the study (n = 358/398).

**Age (years)**		3.7 (3.9)
**Sex (M∶F ratio)**		1.63∶1
**Definitive diagnosis (%)^&^**		75% of cases
**Serogroup (%)#**		
	B	57%#
	C	4%
	not available / not serogrouped	38%
**Ethnicity (%)**		
	Western Europeans	87%
	Other ethnicities or population groups	13%
**Diagnosis**		
	Meningococcemia	50%
	Meningitis	12%
	Both	38%
**Clinical presentation**		
	Septicaemia	59%
	Severe sepsis	11%
	Septic shock	30%
**Clinical Scores**		
	Meningococal Septic Shock Score (MSSS)	1.4 (2.25)
	Glasgow Coma Scale (GCS)	12.9 (2.9)
	PRISM score	9.5 (12.5)
**Organ involvement / Clinical features (%)** *		
	Purpuric rash	81%
	Distal vascular compromise	9%
	DIC	35%
	Cardiovascular dysfunction	45%
	Acute pulmonary lesion	11%
	Acute respiratory distress syndrome	8%
	Neurological dysfunction	24%
	Oligoanuria	18%
	Renal dysfunction	10%
	Hematological dysfunction	35%
	Refractory hypotension	16%
	Liver dysfunction	7.5%
	Purpuric rash	81%
**Outcome**		
	Glasgow Outcome Scale (GOS)	4.8 (0.7)
	Pediatric Overall Performance Category (POPC)	1.2 (0.7)
	Exitus (%)	16 exitus (4%)

& Isolation of *Neisseria meningitidis* from a normally sterile site; OR detection of specific meningococcal DNA sequences in a specimen from a normally sterile site by nucleic acid amplification testing. The rest of subjects fulfilled suggestive criteria (detection of Gram-negative diplococci in Gram's stain of specimen from a normally sterile site or from suspicious skin lesion; or high titre immunoglobulin class M (IgM) or significant rise in IgM or immunoglobulin class G (IgG) titres to outer membrane protein antigens of *N. meningitides.*

# B serogroup accounted for 91.6% of all serogrouped samples.

* Detected anytime during the illness and defined according to Goldstein et al. Pediatr Crit Care Med 2005;6:2–8.

DIC disseminated intravascular coagulation.

Results are expressed as mean (sd) unless otherwise specified.

## Discussion

Recent studies indicate that mitochondrial dysfunction plays a role in the pathogenesis of a number of human diseases. Variation in the mtDNA genome has been suggested as a risk factor in several complex and common multi-factorial diseases. The present study aimed to analyse the potential role of several known mtSNPs as susceptibility factors in MD. We first filtered out from the statistical analysis the non-Spanish patients (excluding also those patients with non-Spanish mothers and/or grandmothers). Patients with a non-European self-reported ancestry or a non-European genetic background, assessed by a set of AIMs in cases and CG2, were also excluded from the study. These two initial criteria were rigorously applied in all patients in order to reduce as much as possible the potential confounding effect of hidden population stratification.

Statistical analysis of cases and CG1 indicated that polymorphism G11719A could be a strong indicator of susceptibility risk to MD. A second round of analyses using an independent series of controls (CG2) could not, however, confirm the initial finding, and instead suggested another mtSNP candidate with a marginal significant *P*-value, namely A4769G. We claim that the most plausible scenario that would explain this ambiguous result is the effect of residual hidden population stratification that could not be ‘erased’ by use of the tools described above because (i) the AIMs employed in the present study were not originally designed to detect differences within European populations and are probably even less efficient at detecting patterns of variation within Iberia; to our knowledge, such an ideal panel able to detect stratification on the geographic scale considered in the present study does not exist; and (ii) it is also possible that the detection of stratification using autosomal SNPs (e.g. Genomic Control) [32], [53], [54] is not at the level of resolution needed to detect population sub-structured patterns in mtDNA variability. Moreover, a proper implementation of a Genomic Control would require the use of various hundreds of autosomal SNPs (especially in markers strongly stratified as it is the case of mtDNA), which contrast with the few SNPs that are usually interrogated in case-control mtDNA studies. The use of AIMs, as done in the present study, could be a reasonable solution at least to detect obvious (genomic) outliers, but a more sensitive set of AIMs would be needed to detect stratification at the level of more homogeneous populations such as those from Spain mainland.

Our study concludes that mtDNA does not seem to play a role in MD susceptibility, course or prognosis; at least from the point of view of the mtSNPs analyzed in the present study and the population samples considered. However, the present study indicates that evidences of positive associations can arise from deficient sampling procedures or a lack of special care in consideration of the potential confounding effects of population sub-structure in mtDNA studies. In our particular study, it was not possible to recruit a second independent collection of cases, owing to the relatively low prevalence of MD; note however that the present study is, to our knowledge, the largest MD cohort analysed to date for a genetic condition. This fact led us to consider a second group of matched controls to replicate the positive signal of association observed in the first control group; however, this time our best candidate mtSNP did not show evidences of association. Therefore, the use of two different control groups from the Iberian Peninsula has allowed us to rule out the presumable association of G11719A with MD. In addition, the use of a moderate approach to correct the undesirable effect of multiple tests in the inflation of type I error has also allowed us to avoid false claims of association of hg U with ARDS, a serious reaction to various forms of injuries to the lung and strongly related to MD patients. Our results also indicate that genotyping bias could also contribute undesirably to a false positive result in mtDNA association studies (see Text S1). Random variation of the test statistics could also explain at least in part the discrepancy observed between CG1 and CG2.

Spurious associations can easily arise from hidden minor levels of population stratification, even after those patients with a different genetic background have been filtered out. The risk of false positives owing to stratification is particularly high in mtDNA studies because the effective population size of this marker is lower than autosomal ones, and therefore mtDNA variability is more deeply structured in human populations. All together, these results suggest that more sensitive AIMs, suitable for detecting stratification within more restricted geographical locations (within the Spanish territory), would be needed in future studies. Special care must be taken in mtDNA association studies of multi-factorial diseases to avoid false claims of association.

## Materials and Methods

### Ethics Statement

The study was conducted according to the Spanish Law for Biomedical Research (Law 14/2007- 3 of July) and complied with the Declaration of Helsinki. The study and the use of archive samples for this project were approved by the Research Ethics Committee of Galicia and the Ethics Committee of the University of Santiago de Compostela. Written informed consent was obtained from all patients. All the samples were collected anonymously.

### Study Subjects

A prospective controlled study performed through a national research network on MD (ESIGEM network – http://www.esigem.org) that includes 41 Spanish paediatric intensive care units was carried out between January 2006 and July 2008.

Cases were 307 paediatric patients with MD; the mean age at diagnosis was 3.7 years (SD: 3.9). More than 200 parameters of clinical interest were obtained from each patient, including demographic, clinical, analytical, and prognostic data. Among these variables we included: (1) clinical data such as diagnosis and clinical presentation, vaccination status, time from starting symptoms to admission, length of stay at the paediatric critical care unit and at the hospital ward, mortality, morbidity, treatment, and also two scores (Paediatric Risk of Mortality Score, and Meningococcal Septic Shock Score); (2) microbiology data such as serogroup, serotype and place of isolation; and (3) laboratory data comprising white and red cells, platelets, glucose, creatine, sodium, potassium, pH, PaO_2_/FiO_2_, calcium, coagulation test and procalcitonine. However, in order to minimise the number of hypotheses tested, only a selection of the 25 most important ones was used for comparison with the hg status of patients (Table 3).

Two different ethnicity-matched control series were used. The first series (CG1) comprised 917 Spanish individuals, including breast cancer patients and controls that were previously employed in [21], [39]; a sub-sample of the CG1 was previously tested for population stratification by means of a panel of neutral autosomal SNPs [40]. The second control series (CG2) included 616 new genotyped Spanish individuals. A third group composed of 597 sporadic breast cancer cases and controls (CG3), collected in the Canary Islands, was additionally used to test some additional hypotheses (see below). Genotypes in the cases and controls series were re-checked by two independent analysts in order to minimise the probability of genotyping errors. Ambiguous calls were eliminated from the analysis.

### mtSNP Selection

All the samples were genotyped for a set of 25 mtSNPs, some of them representing main branches of the European mtDNA phylogeny, such as hg H, U, K, etc.; the panel fully overlapped with the mtSNPs genotyped in [21].

### Ancestry Test

A panel of 34 autosomal AIMs was used to assess ancestry in cases and CG2. These SNPs exhibit highly contrasting allele frequency distributions between major population groups. Assignment of population membership of our Spanish patients into African, Native American, and European ancestry was assessed by adding a sub-set of samples from the CEPH panel (Centre d'Etude du Polymorphisme Humain; http://www.cephb.fr/) as well as additional samples from the *SNPforID* project [41]; all of them were also genotyped for the same 34-plex panel. These samples represented the three main ancestral source populations that would probably have contributed to the present genetic composition of the Spanish population (considering the existence of recent immigrants coming mainly from America and Africa), and in particular to our sample of MD patients. Genotypes were retrieved with the *SNPforID* browser (http://spsmart.cesga.es/snpforid.php; [42]; see also [43]). The final sets of samples used for the inference of individual ancestry were as follows: Africa (*N* = 225), including Biaka Pygmy (Central African Republic; *N* = 23), Mbuti Pygmy (Democratic Republic of Congo; *N* = 13), Bantu-speakers from Kenya (*N* = 11), Mozambique (*N* = 60), San (Namibia; *N* = 6), Yoruba (Nigeria; *N* = 22), Mandenka (Senegal; *N* = 22), Somalia (*N* = 60), South African Bantu (*N* = 8); America (*N* = 208), including Karitiana (Brazil; *N* = 14), Surui (Brazil; *N* = 8), Awa (Colombia; *N* = 38), CEPH-Colombians (*N* = 7), Coiyama (Colombia; *N* = 71), Pijao (Colombia; *N* = 35), Maya (Mexico, *N* = 21), Pima (Mexico, *N* = 14); and Europe (*N* = 278), including Denmark (*N* = 60), French-Basques (France, *N* = 24), France (*N* = 28), Sardinia (Italy, *N* = 28), Tuscany (Italy, *N* = 8), Bergamo (Italy; *N* = 13), Galicians (North-West Spain; *N* = 60), Orkney Islands (Scotland; *N* = 15), Russia (*N* = 25), Adygei (Russian Caucasus; *N* = 17).

STRUCTURE v.2.3.1 [33] was used to estimate the proportion of inferred ancestry of individuals (membership) into Europe, America, and Africa groups. This information was employed to monitor the effect of population stratification in association tests. Runs consisted of 200,000 Markov Chain steps after a burn-in of length 200,000 with five replicates for each *K* value from two to six. The *a posteriori* probability of *K* equal to three was virtually one.

AIMs variability was also analysed by Principal Component Analysis (PCA). A modified version of the R library *SNPassoc* [44] that allows for tri-allelic SNP status was used.

### Minisequencing Genotyping

All the samples were genotyped according to the protocols described in [45], [46] for the mtSNPs and according to [41] for the AIMs. In both cases, the genotyping method was based on a minisequencing SNaPshot approach. MtDNA haplotypes were checked by a phylogenetic-based approach as performed in previous studies [47], [48], [49], [50], [51], in order to minimise the probability of genotyping errors.

### Association Tests

Associations were assessed for individual mtSNPs by comparison of allele frequencies between cases and controls for the two control series separately (CG1 and CG2), and with a one degree of freedom chi-square test, or Fisher's exact test for cell counts below five. A permutation test was used to address the issue of multiple testing in mtSNP association tests. Unconditional logistic regression was used to model the effect of hg status in clinical variables, as well as to model the effect of these variables with respect to ancestry proportions of patients. Multinomial logistic regression was used to test association in the case of clinical variables of multinomial nature.

The statistical packages Stata v.8 (http://www.stata.com/) and R (http://www.r-project.org/) were employed to carry out most of the statistical analysis. Multinomial logistic regression was computed by means of the R library *vglm.*

Power calculations were performed in the Quanto software [52]. Power is computed under allelic principles that do not necessarily fulfil the specific conditions affecting mtDNA variants. There is however no other option available that is specifically designed to compute power in mtDNA case-control disease studies.

## Supporting Information

Text S1 Population sub-structure of mtDNA variants.
(0.29 MB DOC)
