# Supplementary material for: Investigating the Role of Mitochondrial Haplogroups in Genetic Predisposition to Meningococcal Disease
Source: PLoS One. 2009 Dec 17;4(12):e8347. doi: 10.1371/journal.pone.0008347 (PMC2790606; doi:10.1371/journal.pone.0008347)
Supplement: Text S1 — Population sub-structure of mtDNA variants. (0.29 MB DOC) [file pone.0008347.s001.doc]

**Text S1.**

Sub-structure of variant G11719A in the Spanish population and genotyping bias responsible for its positive association with MD

The status of G11719A can be safely imputed from control region data available in the literature from various Spanish regions. This variant is mutationally stable [1] and therefore it characterises the great majority (if not all) of the non-R0 mtDNAs (see phylogeny in Figure 1). The average mainland Spanish (inferred) frequency of G11719A is ~46.0%; this value is close to the frequency of G11719A in our sample of cases (49.6%). The frequency of G11719A was, however, unusually lower in CG1 (37.7%), the control group where this variant shows up as positively associated with the MD patients. As shown in the map in Figure 1, only the population from the Basque country has a similar very low (inferred) frequency for this variant, mainly owing to the high prevalence of haplogroup V and several H sub-lineages (all of them nested within R0 and therefore characterised by 11719G) [2,3,4]. The frequency of G11719A in CG2 is 47.4% as it corresponds with the average frequency in Spanish mainland (Figure 1). It is also important to highlight the fact that the population (inferred) frequency of G11719A in the Canary Islands is significantly higher (59.1%) than in the mainland, and it is even higher in the Balearic Islands (62.0%), with an extreme frequency peak in the Balearic Chuetas (72.9%).

We further explored the causes of the unusual frequency of CG1 by re-examining the genotyping calling rate of G11719A. The percentage of missing data at this polymorphism was extremely high in CG1 (25%), but also in CG2 (17%), obviously indicating some problem affecting the efficiency of this particular minisequencing probe. The haplogroup status of the samples bearing missing calls for G11719A could be perfectly inferred from the remaining mtSNP data, indicating that the inferred frequency of G11719A in CG1 is ~42%, still well below the average frequency of this variant in mainland Spain and below cases. Therefore, genotyping bias explains only partially the difference in frequency observed between cases and CG1.

Given the population context described above, it is straightforward to simulate a scenario of population sub-structure between cases and controls by selecting a third Spanish control group from the Canary Islands; these islands were probably founded about ~2.2-2.4 kya [1,5] and we already know that this population has a slightly different mtDNA background from other mainland population groups (e.g. [4,6,7,8,9,10]). Thus, we compared our MD series with a third series of Spanish controls (CG3; power to detect OR>1.6 is 88%; assuming MAF = 20%) (see [11]). When comparing cases and CG3, we observed several mtSNPs, including 11719 spuriously associated with MD (data not shown). This result is concordant with the inferred population frequency observed in previous studies for G11719A (Figure 1).

Other considerations regarding population substructure in mtDNA association studies

Another way to simulate the confounding effect of population stratification on the association test is to add to the statistical analysis the small number of MD gypsies and/or other patients that were initially ruled out owing to their dubious European ancestry or self-reported non-Spanish origin. Again, the impact of these patients on the association test was very strong, giving rise to a number of new statistical significant associations (data not shown). This finding supports the idea that the addition of a small number of patients with different genetic background to the study might be a common risk factor for type I error.

There are other factors that should also be considered in the evaluation of signals of positive associations. For instance, simulation analysis has demonstrated that large cohorts are required for reliable detection of an mtDNA association with complex human disease [12]. Large cohorts are however extremely unusual in mtDNA studies. On the other hand, the benefit provided by the use of large cohorts in increasing the power to detect association is counterbalanced by the fact that large sample sizes also inflate the confounding effect of population stratification [13,14,15,16,17,18].

Last but not least, the association of any mtSNP with a particular disease should also be consistent with other evidences. For instance, Pezzotti and colleagues [19] claimed that the A10398G polymorphism plays a role in interaction with alcohol consumption and breast cancer risk. This variant makes up about a quarter of a typical European population and appears in parallel in several branches of the mtDNA tree (e.g. haplogroup J or K1, see Figure 1). It is therefore not clear if the association observed by Pezzotti is because of A10398G or some of the sub-branches defined by this variant, as it was also recognised by the authors. Nevertheless, the strongest evidence in favour of their positive result of association (note that the authors could not replicate this association in a different cohort analysed in the same study) is the fact that other available studies on breast cancer patients also observed similar positive associations, e.g. [20]; it was not mentioned however that the referred supporting studies were also critically questioned [11]. Another caveat that should also be taken into account when we consider the potential pathogenic role of an mtSNP is that the non-synonymous status of a variant might be meaningless. For instance, if we look at the complete genomes of healthy individuals in the 241 complete genomes analysed by Coble and colleagues [21], there are a total of 117 different variants that are non-synonymous, including A10398G.

Yang et al. [22] claimed that haplogroup R predicts survival advantage in severe sepsis in the Han population. The authors stated (p. 188) that ‘*In all comparisons, P < 0.05 was considered statistically significant*’. The best *p*-value observed in this study was 0.001 for the mtSNP defining haplogroup R, C12705T. The fact that this *P*-value was not corrected for multiple tests (e.g. 11 mtSNPs were genotyped coupled with other polymorphisms observed by sequencing the control region) and the fact that there was no control for the confounding effect of potential population sub-structure in their Han population sample are good reasons to believe that this finding is also a false positive.

Given all these caveats, the study of Baudouin and colleagues [23] suggesting the presumable association of haplogroup H in survival after sepsis should be viewed with caution pending a confirmatory study on a replicating sample.

Finally, Mueller et al. [24] have recently re-evaluated the potential role of mitochondrial haplogroups in prostate cancer in middle European ‘Caucasians’. These authors could not replicate previous surveys on prostate cancer [25]. According to Mueller and colleagues, previous positive findings could be due to statistical deficiencies and problems related to the selection of control individuals, as also discussed in the present study. Their criticisms regarding the potential positive association of haplogroup U in cancer are also supported by the study of Mosquera-Miguel et al. [11].

References

1. Soares P, Ermini L, Thomson N, Mormina M, Rito T, et al. (2009) Correcting for purifying selection: an improved human mitochondrial molecular clock. Am J Hum Genet 84: 740-759.

2. Achilli A, Rengo C, Magri C, Battaglia V, Olivieri A, et al. (2004) The molecular dissection of mtDNA haplogroup H confirms that the Franco-Cantabrian glacial refuge was a major source for the European gene pool. Am J Hum Genet 75: 910-918.

3. Torroni A, Bandelt H-J, Macaulay V, Richards M, Cruciani F, et al. (2001) A signal, from human mtDNA, of postglacial recolonization in Europe. Am J Hum Genet 69: 844-852.

4. Álvarez-Iglesias V, Mosquera-Miguel A, Cerezo M, Quintáns B, Zarrabeitia MT, et al. (2009) New population and phylogenetic features of the internal variation within mitochondrial DNA macro-haplogroup R0. PLoS ONE 4: e5112.

5. Galván Santos B, Hernández Gómez CM, Alberto Barroso V, Barro A, Eugenio CM, et al. (1999) Poblamiento prehistórico en la costa de Buena Vista del Norte (Tenerife). Investigaciones Arqueológicas en Canarias 6: 9–258.

6. Salas A, Comas D, Lareu MV, Bertranpetit J, Carracedo Á (1998) mtDNA analysis of the Galician population: a genetic edge of European variation. Eur J Hum Genet 6: 365-375.

7. Salas A, Lareu V, Calafell F, Bertranpetit J, Carracedo A (2000) mtDNA hypervariable region II (HVII) sequences in human evolution studies. Eur J Hum Genet 8: 964-974.

8. Salas A, Richards M, De la Fé T, Lareu MV, Sobrino B, et al. (2002) The making of the African mtDNA landscape. Am J Hum Genet 71: 1082-1111.

9. Crespillo M, Luque JA, Paredes M, Fernández R, Ramirez E, et al. (2000) Mitochondrial DNA sequences for 118 individuals from northeastern Spain. Int J Legal Med 114: 130-132.

10. Rando JC, Cabrera VM, Larruga JM, Hernández M, González AM, et al. (1999) Phylogeographic patterns of mtDNA reflecting the colonization of the Canary Islands. Ann Hum Genet 63: 413-428.

11. Mosquera-Miguel A, Álvarez-Iglesias V, Vega A, Milne R, Cabrera de León A, et al. (2008) Is mitochondrial DNA variation associated with sporadic breast cancer risk? Cancer Res 68: 623-625.

12. Samuels DC, Carothers AD, Horton R, Chinnery PF (2006) The power to detect disease associations with mitochondrial DNA haplogroups. Am J Hum Genet 78: 713-720.

13. Marchini J, Cardon LR, Phillips MS, Donnelly P (2004) The effects of human population structure on large genetic association studies. Nat Genet 36: 512-517.

14. Pritchard JK, Rosenberg NA (1999) Use of unlinked genetic markers to detect population stratification in association studies. Am J Hum Genet 65: 220-228.

15. Pritchard JK, Stephens M, Donnelly P (2000) Inference of population structure using multilocus genotype data. Genetics 155: 945-959.

16. Devlin B, Bacanu SA, Roeder K (2004) Genomic control to the extreme. Nat Genet 36: 1129-1130; author reply 1131.

17. Devlin B, Roeder K, Bacanu SA (2001) Unbiased methods for population-based association studies. Genet Epidemiol 21: 273-284.

18. Devlin B, Roeder K, Wasserman L (2001) Genomic control, a new approach to genetic-based association studies. Theor Popul Biol 60: 155-166.

19. Pezzotti A, Kraft P, Hankinson SE, Hunter DJ, Buring J, et al. (2009) The mitochondrial A10398G polymorphism, interaction with alcohol consumption, and breast cancer risk. PLoS One 4: e5356.

20. Bai RK, Leal SM, Covarrubias D, Liu A, Wong LJ (2007) Mitochondrial genetic background modifies breast cancer risk. Cancer Res 67: 4687-4694.

21. Coble MD, Just RS, O'Callaghan JE, Letmanyi IH, Peterson CT, et al. (2004) Single nucleotide polymorphisms over the entire mtDNA genome that increase the power of forensic testing in Caucasians. Int J Legal Med 118: 137-146.

22. Yang Y, Shou Z, Zhang P, He Q, Xiao H, et al. (2008) Mitochondrial DNA haplogroup R predicts survival advantage in severe sepsis in the Han population. Genet Med 10: 187-192.

23. Baudouin SV, Saunders D, Tiangyou W, Elson JL, Poynter J, et al. (2005) Mitochondrial DNA and survival after sepsis: a prospective study. Lancet 366: 2118-2121.

24. Mueller EE, Eder W, Mayr JA, Paulweber B, Sperl W, et al. (2009) Mitochondrial haplogroups and control region polymorphisms are not associated with prostate cancer in Middle European Caucasians. PLoS One 4: e6370.

25. Booker LM, Habermacher GM, Jessie BC, Sun QC, Baumann AK, et al. (2006) North American white mitochondrial haplogroups in prostate and renal cancer. J Urol 175: 468-472; discussion 472-463.

26. González AM, Brehm A, Pérez JA, Maca-Meyer N, Flores C, et al. (2003) Mitochondrial DNA affinities at the Atlantic fringe of Europe. Am J Phys Anthropol 120: 391-404.

27. Maca-Meyer N, Sánchez-Velasco P, Flores C, Larruga JM, González AM, et al. (2003) Y chromosome and mitochondrial DNA characterization of Pasiegos, a human isolate from Cantabria (Spain). Ann Hum Genet 67: 329-339.

28. Bertranpetit J, Sala J, Calafell F, Underhill PA, Moral P, et al. (1995) Human mitochondrial DNA variation and the origin of Basques. Ann Hum Genet 59: 63-81.

29. Côrte-Real HBSM, Macaulay VA, Richards MB, Hariti G, Issad MS, et al. (1996) Genetic diversity in the Iberian peninsula determined from mitochondrial sequence analysis. Ann Hum Genet 60: 331-350.

30. Richards M, Côrte-Real H, Forster P, Macaulay V, Wilkinson-Herbots H, et al. (1996) Paleolithic and neolithic lineages in the European mitochondrial gene pool. Am J Hum Genet 59: 185-203.

31. Richards M, Macaulay V, Hickey E, Vega E, Sykes B, et al. (2000) Tracing European founder lineages in the Near Eastern mtDNA pool. Am J Hum Genet 67: 1251-1276.

32. Plaza S, Calafell F, Helal A, Bouzerna N, Lefranc G, et al. (2003) Joining the pillars of Hercules: mtDNA sequences show multidirectional gene flow in the western Mediterranean. Ann Hum Genet 67: 312-328.

33. Larruga JM, Diez F, Pinto FM, Flores C, Gonzalez AM (2001) Mitochondrial DNA characterisation of European isolates: the Maragatos from Spain. Eur J Hum Genet 9: 708-716.

34. Picornell A, Gomez-Barbeito L, Tomas C, Castro JA, Ramon MM (2005) Mitochondrial DNA HVRI variation in Balearic populations. Am J Phys Anthropol 128: 119-130.

35. Falchi A, Giovannoni L, Calo CM, Piras IS, Moral P, et al. (2006) Genetic history of some western Mediterranean human isolates through mtDNA HVR1 polymorphisms. J Hum Genet 51: 9-14.

**Figure 1.** The map in the top shows the frequency estimates for the G11719A variant in different Spanish locations. The codes for the regions and sample sizes are as follows: [1] Galicia (*N* = 418; [4,6,7,26]); [2] Cantabria (*N* = 99; [4,27]); [3] Basque Country (*N* = 172; [28,29,30,31]); [4] Catalonia (*N* = 280; [4,9,29,32]); [5] Maragatos from León (*N* = 49; [33]); [6] Castilla (*N* = 38; [33]); [7] Valencia (*N* = 42; [34]); [8] Andalucía (*N* = 601; unpublished data and [29,32,33,35]); [9] Balearic Islands (*N* = 141; [34]) ; [10] Chuetas from Balearic Islands (*N* = 48; [34]); [11] Canary Islands (*N* = 308; [10]). The phylogenetic tree in the bottom indicates the main phylogenetic branches represented by the mtSNPs genotyped in the present study (with the exception of mtSNP T16519C because it is not diagnostic of any branch). Some of them are redundant in the worldwide tree and appear sporadically in parallel in other (sub)branches (e.g. G3010A in J1, T4336C in U6d, G8994A in H21, G13708A in K2a3, H18 and X2b/X2d, etc). The dotted green rectangle indicates the part of the tree that carries 11719G while the dotted red rectangle groups the branches with 11719A. The bottom left legend indicates the functional status of mtSNPs.

**Figure 1.**
